# Effects of grain adaptation programs and antimicrobial feed additives on performance and nutrient digestibility of *Bos indicus* cattle fed whole shelled corn

**DOI:** 10.1093/tas/txab119

**Published:** 2021-07-13

**Authors:** Andrea M Mobiglia, Fernando R Camilo, Victor R M Couto, Flavio G F Castro, James S Drouillard, Vinícius N Gouvêa, Juliano J R Fernandes

**Affiliations:** 1 Departamento de Zootecnia, Universidade Federal de Goiás, Goiânia, Goiás 74690–900, Brazil; 2 Agrocria Ind. & Com. LTDA, Goiânia, Goiás 74513-050, Brazil; 3 Department of Animal Science and Industry, Kansas State University, Manhattan, KS 66506, USA; 4 Department of Animal and Range Sciences, New Mexico State University, Clayton Livestock Research Center, Clayton, NM 88415, USA

**Keywords:** adaptation, beef cattle, feedlot, monensin, roughage, virginiamycin

## Abstract

Two experiments were conducted to evaluate the effects of feed additives [monensin (**MON**); 30 mg/kg of dry matter (**DM**), and virginiamycin (**VM**); 25 mg/kg DM] and grain adaptation programs [adding roughage (**ROU**; sugarcane bagasse) or not (**NO-ROU**) during the 20-d adaptation period] on performance, carcass characteristics, and nutrient digestibility of *Bos indicus* cattle fed finishing diets containing 85% whole shelled corn and 15% of a pelleted protein-mineral-vitamin supplement. In Exp.1, 105 Nellore bulls [initial body weight (**BW**) = 368 ± 25 kg] were used in a complete randomized block design with a 2 × 2 factorial arrangement of treatments, consisting of two feed additives (MON and VM) associated with two adaptation programs (ROU or NO-ROU during the 20-d adaptation period). Effects of feed additives × adaptation programs were not detected (*P* ≥ 0.13). Feed additives did not affect dry matter intake (**DMI**), average daily gain (**ADG**), and feed efficiency (**G:F**) during the 20-d adaptation period (*P* ≥ 0.35). During the total feeding period (105 d), feeding MON decreased DMI (*P* ≤ 0.03) compared to VM. Adding sugarcane bagasse to finishing diets during the 20-d adaptation period (ROU) increased ADG (*P* = 0.05) and G:F (*P* = 0.03), and tended to increase BW (*P* = 0.09) compared to NO-ROU. In Exp. 2, 10 ruminally cannulated Nellore steers (BW = 268 ± 38 kg) were used in a completely randomized design to evaluate the effects of the two feed additives used in the Exp. 1 (MON and VM; 5 steers/treatment) on DMI, total apparent digestibility of nutrients, and ruminal fermentation characteristics. No differences in DMI, total tract apparent digestibility of nutrients, and ruminal fermentation characteristics were observed between MON and VM (*P* ≥ 0.32). An effect of sampling day (*P* < 0.001) was observed for ruminal pH, which was greater on day 0 compared to day 7, 14, and 21 of the experimental period (*P* ≤ 0.05). In summary, supplementing monensin and virginiamycin for finishing Nellore bulls fed whole shelled corn diets, resulted in similar growth performance and carcass characteristics. Including sugarcane bagasse to adapt finishing bulls to no-roughage diets containing whole shelled corn is an alternative to increase growth performance.

## INTRODUCTION

Roughage in feedlot diets is one of the most expensive ingredients on an energy basis (Bartle and Preston, 1991), and is traditionally included at low percentage in high-concentrate diets to maintain rumen health and maximize dry matter intake (**DMI**; Galyean and Defoor, 2003). According to Turgeon et al. (2010), although removal of roughage from finishing diets has management and economic advantages, no-roughage diets must be formulated carefully to avoid digestive disorders and to optimize growth performance.

Extensive methods of grain processing, such as steam flaking (**SFC**), markedly increase the rate and extent of ruminal starch digestion and cattle growth performance (Zinn et al., 2007; Owens and Basalan, 2013; Gouvêa et al., 2016). On the other hand, feeding whole shelled corn (**WC**) resulted in similar growth performance compared to ground corn (Gorocica-Buenfil and Loerch, 2005). According to Turgeon et al. (2010), feeding WC would be one approach for eliminating roughage from finishing diets because of the slower rate and extent of ruminal starch digestion of WC compared to SFC or high-moisture corn. However, information on how to adapt feedlot cattle to no-roughage WC-based diets is lacking.

Marques et al. (2016) observed that adding roughage to WC-based diets increased growth performance of finishing bulls compared to no-roughage WC-based diets, due to the increase in DMI and net energy (**NE**) intake. However, the trial was preceded by a 21-d adaptation period, during which roughage was reduced from 30% to 15% of the diet (DM basis), and no data of the adaptation period was presented by these authors.

Rumen fermentation characteristics and DMI of feedlot cattle can also be affected by feeding certain feed additives (Butaye et al., 2003; Duffield et al., 2012). Antimicrobial feed additives have been studied over 60 yr in animal nutrition for manipulation of ruminal fermentation (Visek, 1978). Monensin is the most common ionophore used in finishing diets in the USA (Samuelson et al., 2016). Also, according to Pinto and Millen (2019) ionophores are the primary feed additives used in finishing diets in Brazil. In a meta-analysis conducted by Duffield et al. (2012), monensin decreased DMI by 3% and improved feed efficiency of finishing beef cattle by 2.5% to 3.5%. Virginiamycin is a non-ionophore antibiotic that inhibits growth of Gram-positive bacteria (Cocito, 1979) and has the potential of enhance growth performance and reduce the incidence of liver abscesses in finishing cattle, without affecting DMI (Rogers et al., 1995).

Based on the aforementioned, we hypothesized that growth performance of finishing cattle fed WC-based diets would be improved if either roughage during adaptation period or virginiamycin is added to the diet. Thus, the objective of this experiment was to evaluate the effects of grain adaptation programs and feed additives on growth performance, carcass characteristics, and nutrient digestibility of *Bos indicus* cattle fed diets containing 85% whole shelled corn and 15% of a pelleted protein-mineral-vitamin supplement.

## MATERALS AND METHODS

Experiments 1 and 2 were conducted at the Agrocria Research Center and at the Experimental Feedlot Research facility of the School of Veterinary Medicine and Animal Science of the Federal University of Goiás, in Goiânia, state of Goiás, Brazil, respectively. All animals were cared for in accordance with experimental protocols approved by the Institutional Animal Care and Use Committee of the Federal University of Goiás (protocol number # 087/14).

### Experiment 1. Animal Performance Animals, Housing, and Experimental Design

A total of 105 commercial Nellore bulls [*Bos indicus*; initial shrunk body weight (**BW**) = 368 ± 25 kg] were used in this experiment. Bulls were raised in continuous grazing systems since birth, but no previous nutritional or management history was available. Upon feedlot arrival bulls were individually weighed (idBECK 3.0, Beckhauser, Maringá, Paraná, Brazil) vaccinated against clostridiosis (5 mL s.c.; Poli-Star, MSD Saúde Animal, São Paulo, Brazil), treated for external and internal parasites with 3.15% ivermectin (5 mL s.c.; Ivomec Gold, Boehringer-Ingelheim, São Paulo, Brazil) and identified with a unique ear tag. Bulls were blocked by initial BW (5 weight blocks), assigned to 20 pens, and pens within weight block were randomly assigned to treatments (5 pens/treatment). Five bulls (1 bull/weight block) were slaughtered at the beginning of the experiment to estimate the initial hot carcass weight (**IHCW**; equation 1). The remaining bulls (*n* = 100) were allocated to 20 covered pens (15 × 20 m; 5 bulls/pen and 0.57 m of linear bunk space/bull) in a randomized complete block design with 2 × 2 factorial arrangement of treatments consisting of two feed additives [monensin (**MON**; 30 mg/kg dry matter; DM) or virginiamycin (**VM**; 25 mg/kg DM)] and two adaptation programs [adding (**ROU**) or not roughage (**NO-ROU**; sugarcane bagasse) during the 20-d adaptation period]. The length of the adaptation period was based on Marques et al. (2016) that also fed finishing Nellore bulls with WC-based diets.


$$\begin{array}{l} IHCW=(IBW\times 0.4646)+25.84\;(R^{2}=0.93) \end{array}$$ (1)

where, IHCW = initial hot carcass weight; **IBW**= initial body weight.

### Feeding Management

Bulls were fed once daily (0700 h) and had *ad libitum* access to feed and fresh water throughout the trial (105 days feeding period). Bulls fed NO-ROU were fed the basal total mixed ration (**TMR**; Table 1) containing 85% of WC and 15% pelleted protein, mineral, and vitamin supplement (DM basis; Engordim GI, Agrocria Nutrição Animal e Sementes, Goiânia, Goiás, Brazil) since the first day of the experiment. The TMR was formulated to meet the requirements of finishing cattle for 1.4 kg average daily gain (**ADG**) as specified by the NASEM (2016). Bulls fed ROU were progressively stepped up to NO-ROU by decreasing the dietary concentration of sugarcane bagasse (**SCB**) every 5 days from 40% to 30% to 20 to 10% (DM basis) during the first 20-d of the experiment (adaptation period; Table 1). The SCB was daily and manually mixed to the pelleted supplement before feeding. At the end of the 20-d adaptation period, bulls fed ROU were then switched to the NO-ROU diet.

**Table 1.** Ingredient and chemical composition of the basal diet used in Exp. 1 and 2 [dry matter (DM) basis]

Item	Step-up diets				Finishing det
	1	2	3	4	
Ingredient, % of DM					
Sugarcane bagasse	40.0	30.0	20.0	10.0	-
Whole shelled flint corn	45.0	55.0	65.0	75.0	85.0
Pelleted supplement^1^,^2^	15.0	15.0	15.0	15.0	15.0
Analyzed composition^3^					
Dry matter, %	71.1	76.6	82.1	87.6	93.1
Crude protein, %	11.3	12.1	12.7	13.2	13.6
Neutral detergent fiber, %	37.7	31.2	24.8	17.8	12.0
Ether extract, %	2.60	2.74	2.89	3.03	3.19
Ash, %	5.50	5.12	4.75	4.38	4.07
Starch, %	32.4	39.4	46.7	53.7	60.3
Total digestible nutrients, %^4^	49.5	58.0	66.4	74.9	83.3
Net energy of maintenance, Mcal/kg^5^	0.95	1.24	1.51	1.78	2.04
Net energy of gain, Mcal/kg^5^	0.40	0.67	0.92	1.16	1.38

^1^ Feed additives in Exp. 1 and Exp. 2 [monensin (30 mg/kg DM; Rumensin-200; Elanco Saúde Animal, São Paulo, São Paulo, Brazil) or virginiamycin (25 mg/kg DM; Phigrow; Phibro Animal Health Corporation, Guarulhos, São Paulo, Brazil)] were added to the pelleted supplement that were produced at a commercial feed mill (Agrocria Nutrição Animal e Sementes, Anápolis, Goiás, Brazil).

^2^ Composition: 100 g/kg moisture; 380 g/kg crude protein; 200 g/kg ash; 12 g/kg ether extract; 220 g/kg acid detergent fiber; 42 g/kg Ca; 6000 mg/kg P; 9.7 mg/kg Na; 15 g/kg K; 3000 mg/kg Mg; 420 mg/kg Zn; 180 mg/kg Mn; 175 mg/kg Cu; 24 mg/kg F; 5 mg/kg Co; 5 mg/kg I; 4.5 mg/kg S; 1.8 mg/kg Se; 1.4 mg/kg Cr; 0.35 mg/kg Mb; 0.3 mg/kg; 21,000 IU/kg vitamin A; 3,000 IU/kg vitamin D; and 135 IU/kg vitamin E.

^3^ Based on chemical analysis of ingredients collected throughout the experiment (*n* = 7 samples).

^4^ The total digestible nutrients (TDN) were calculated according to the equations described by Weiss et al. (1992) using the processing factor of 0.95 for whole shelled corn (NRC, 2001).

^5^ Calculated from TDN values, assuming that 1 kg TDN = 4.4 Mcal of digestible energy (DE) and the ratio of metabolizable energy (ME) to DE is 0.82. Relationships for converting ME values to net energy of maintenance (NE_m_) and net energy of gain (NE_g_) were according Garrett (1980).

The feed additives [MON (Rumensin-200; Elanco Saúde Animal, São Paulo, São Paulo, Brazil) or VM (Phigrow; Phibro Animal Health Corporation, Guarulhos, São Paulo, Brazil)] were included in the pelleted supplements that were produced at a commercial feed mill following the manufacturing standards for quality and guaranteed levels (Agrocria Nutrição Animal e Sementes, Anápolis, Goiás, Brazil), and were offered to bulls throughout the trial (105 days feeding period). Feed bunks were evaluated daily at 0600 and 1800 h and feed delivery was adjusted so that bunks contained trace amounts of feed at 0700 h. Orts were removed and weighed daily to measure DMI.

The feed ingredients were sampled every other week and analyzed by wet chemistry for nutrient composition [corn composition (DM basis): 92.45 % DM, 9.78 % crude protein (**CP**), 3.4 % ether extract (**EE**), 1.19 % ash, 9.51 % neutral detergent fiber with ash correction (**NDFa**); pelleted supplement (DM basis): 96.51 % DM, 35.29 % CP, 2.0 % EE, 20.37 % ash, and 26.2 % NDFa, 4 mm pellet diameter]. Whole corn used in this study was flint corn, as is commonly used in Brazil (Correa et al., 2002; Gouvêa et al., 2016; Pinto and Millen, 2019). The pelleted supplement was a commercial formulation containing soybean hulls, soybean meal, urea, minerals, and vitamins [nutrient composition: 100 g/kg moisture; 380 g/kg CP; 200 g/kg ash; 12 g/kg EE, and 220 g/kg acid detergent fiber (**ADF**)]. The TMR’s containing 85% of corn and 15% of pelleted supplement containing the feed additives (MON or VM) were also produced at the commercial feed mill (Agrocria Nutrição Animal e Sementes), packed into 40-kg polypropylene bags, and properly labeled for each treatment. The TMR’s were delivered at the feedlot facility at the beginning of the trial and stored in a covered shed. The SCB was stored outside the feedlot facility, and covered with a fabric tarp. The SCB was sampled twice a week and composited for nutrient analysis [nutrient composition (DM basis): 37.48 % DM, 3.28 % CP, 1.96 % EE, 4.88 % ash, 73.69 % NDFa, on DM basis].

Bulls were individually weighed after 16 h of fasting (feed and water) on day 0, after adaptation (20 d), and before harvest (105 d). On 105 d, bulls were transported approximately 15 km in a commercial trailer and were slaughtered on the following day. The hot carcass weight (**HCW**) was obtained at the time of slaughter after exsanguination and removal of head, feet, hides, and viscera (including kidneys and kidney, pelvic and heart fat.

### Sampling, Laboratory Analysis, and Measurements

Diets and feed ingredients were sampled every 15 days and stored at −20°C. Orts were recovered daily and used to determine the DM in an oven at 105^o^C for 24 [AOAC (1995); method: 930.15]. At the end of the trial diets and feed samples were thawed, dried at 55^o^C for 72 h, and ground through a 1-mm screen using a Wiley mill (Tecnal TE-650, Tecnal Equipamentos Científicos, Piracicaba, São Paulo, Brazil). Samples were analyzed for dry matter (DM; method 934.01; AOAC, 1995) and ash (method 942.05; AOAC, 1995). Nitrogen content was determined using a micro Kjeldahl apparatus (TE-036/1 model, Tecnal, Piracicaba, SP, Brazil) according to AOAC (1995; method 976.05). The CP content was calculated by multiplying nitrogen content by 6.25. The EE was determined according to AOAC (1995; method 920.39) using the TE-044 extractor (Tecnal, Piracicaba, SP, Brazil). Sequential detergent fiber analyses (NDF and ADF) was determined according to Van Soest et al. (1991) using the TE-149 Fiber Analyzer (Tecnal, Piracicaba, SP, Brazil). The NDF was determined using heat-stable α-amylase (A3306, Sigma Chemical. Co., St. Louis, MO) without sodium sulfite, and ash and protein-corrected (Licitra et al., 1996). Starch content was analyzed following the methodology described by Bach Knudsen (1997). Total digestible nutrients (**TDN**) of the ingredients were estimated according to Weiss et al. (1992), using presumed processing adjustment factor of 0.95 for whole corn. Net energy of maintenance (**NE**_**m**_) and net energy of gain (**NE**_**g**_) were calculated from TDN value, assuming that 1 kg TDN = 4.4 Mcal of digestible energy (**DE**) and the ratio of metabolizable energy (**ME**) to DE is 0.82. Relationships for converting ME values to NE_m_ and NE_m_ were according Garrett (1980). The ADG, gain-to-feed ratio (**G:F**), and DMI were calculated for each period evaluated. Hot carcass weight (**HCW**) was recorded to calculated dressing percentage (**DP**) by dividing HCW by final BW on 105 d, then multiple by 100.

### Statistical Analysis

Data were analyzed using the “easyanova” package (version 4.0, 2014) of R Software (R Development Core Team, version 3.3.1, 2015) as a randomized complete block design with a 2 × 2 factorial arrangement of treatments [two feed additives (MON and VM)] and two adaptation programs [adding (ROU) or not roughage (NO-ROU) during the 20-d adaptation period]. Pen was the experimental unit and the Kenward–Roger approximation method was used to determine the correct denominator degrees of freedom for testing fixed effects. The model included the fixed effect of feed additives, adaptation program, and feed additives × adaptation program interaction. Pen (feed additives × adaptation program × block) and bull (pen) were used as random variables for all variables analyzed, except for DMI and G:F that did not include bull (pen). When no significant interactions were detected, main effects of feed additives and adaptation programs were evaluated. When significant interactions (*P* < 0.05) were observed for a trait, treatments (feed additives × adaptation program interaction) were compared using Tukey–Kramer test. Results were presented as least-squares means. Statistical differences were considered at *P* < 0.05 and trends were discussed at *P* ≤ 0.10.

### Experiment 2. Digestibility and Ruminal Fermentation Characteristics Animals, Housing, and Experimental Design

Ten ruminally cannulated Nellore steers (*Bos indicus*; BW = 268 ± 38 kg) were used to evaluate the effects of the two feed additives used in Exp. 1 [monensin (MON; 30 mg/kg DM) or virginiamycin (VM; 25 mg/kg DM)] in a completely randomized design with 5 replicates/treatment. Steers were housed in individual pens (12.5 m^2^) with a solid roof and concrete floor and given free choice access to water during the experiment. Vaccination protocol was the same described for Exp. 1.

### Feeding Management

Steers were fed twice a day [0700 h (60% of the feed call) and 1700 h (40% of the feed call)] during 26 days, including 20 days for adaptation to diets and 6 days for sampling and data collection. The basal TMR (Table 1) contained 85% of whole shelled corn and 15% pelleted protein, mineral, and vitamin supplement (DM basis; Engordim GI, Agrocria Nutrição Animal e Sementes, Goiânia, Goiás, Brazil). The TMR was offered to the animals since the first day of the experiment, starting at 1.5% BW and increased (300 g of DM/head/day) until animals reached the *ad libitum* intake (5% orts).

### Sample Collection and Calculations

The amount of feed offered and refusals were recorded and sampled from day 24 to day 26 for DMI calculation. Samples were analyzed as described in Exp. 1. Chromic oxide (5.0 g/dose) was used as an indigestible marker for estimating fecal output and was dosed intraruminally at 0700 and 1900 h daily from days 20 to 26. Fecal samples were manually collected from days 24 to 26 from the rectum of each steer at 0600 h, 1300 h, and 1800 h. Fecal samples were composited by day and analyzed for DM, CP, ash, NDF, and starch as described in Exp. 1. Chromium concentration was analyzed using atomic absorption spectrophotometry as described by Williams et al. (1962). Apparent total tract digestibility of DM, organic matter (**OM**), CP, NDF, and starch were calculated using the equation: apparent total tract digestibility (%) = ([nutrient concentration of feed – nutrient concentration of feces]/nutrient concentration of feed) × 100.

Ruminal fluid was manually collected from the cranial, ventral, and caudal areas of the rumen on days 7, 14, and 21 before feeding (T0) and 3 h, 6 h, 12 h, and 18 h post feeding. Ruminal fluid samples were squeezed through four layers of cheesecloth and the ruminal pH was immediately measured using a portable pH meter (Blue-Top, Bel Equipamentos Analíticos, Piracicaba, São Paulo, Brazil) previously calibrated with buffer solutions of pH 4 and 7. Samples were then composited by day, and one subsample of 50 mL of ruminal fluid was acidified with 0.5 mL sulfuric acid (1:1 ratio), frozen at −20^o^C, and used for N-NH_3_ analysis. Another subsample was stored in 50-mL falcon tubes without preservatives, and frozen at −20^o^C until analysis of volatile fatty acids (**VFA)**. The N-NH_3_ concentration was measured by spectrophotometry (Biospectro SP-22; Biospectro, Curitiba, Paraná, Brazil) at 550 nm according to Chaney and Marbach (1962). The VFA concentrations were analyzed by gas chromatography (Shimadzu GC-2010; Shimadzu Corporation, Kyoto, Japan) using a capillary column (RTX-WAX, 30 m × 0.32 mm ID, 0.25 μm), flame ionization detector and helium (UHP) as carrier gas, according to Palmquist and Conrad (1971).

### Statistical Analysis

Data were analyzed using the “easyanova” package (version 4.0, 2014) of R Software (R Development Core Team, version 3.3.1, 2015) as completely randomized design with two treatments (MON and VM). Animal was the experimental unit. The model included the fixed effect of feed additives and animal as a random effect. Ruminal fermentation characteristics (ruminal pH, VFA, and N-NH_3_) were analyzed as repeated measures over the time, and the model included the fixed effect of treatment, sampling day, and the interaction between treatment × sampling day. The covariance structure type used was compound symmetry because it had the smallest value for Akaike’s information criterion. Since no interactions between treatment × sampling day were observed, this statement was dropped off the model and averages across 24 h periods were used for statistical analysis to simplify data interpretation and reporting. When no significant interactions were detected, the main effects of feed additives and time were evaluated. When significant interactions (*P* < 0.05) were observed for a trait, feed additives were compared within each collection day. Statistical differences were considered at *P* < 0.05 and trends were discussed at *P* ≤ 0.10. Means were presented as least squares means.

## RESULTS AND DISCUSSION

Effects of feed additives × adaptation programs were not detected (*P* ≥ 0.13) in Exp. 1 (Table 2). Feed additives did not affect the DMI, ADG, and G:F during the 20-d adaptation period (*P* ≥ 0.35; Table 2). During the total feeding period (105 d) feeding MON decreased the DMI (*P* ≤ 0.03) compared to VM (Table 2). In a meta-analysis conducted by Duffield et al. (2012) MON decreased DMI an average of 3% but improved feed efficiency of finishing beef cattle by 2.5% to 3.5%. According to the NASEM (2016), ionophores might have negative effects on DMI in receiving cattle. On the other hand, supplementing VM (19–27 mg/kg of DM) enhanced the ADG by 4.6% and the G:F by 3.6%, without affecting DMI (Rogers et al., 1995). Thus, it is reasonable to hypothesize that feeding VM during the feedlot adaptation period would increase growth performance compared to MON. Contrary to our hypothesis, no differences were observed between MON and VM during the 20-d adaptation period, although MON decreased DMI in 8.5% compared to VM during the total feeding period, without affecting G:F. Montano et al. (2015) did not observe any differences between MON (34 mg/kg of DM) and VM (26 mg/kg of DM) on DMI, ADG, and G:F, of finishing cattle, although both feed additives increased G:F compared to the control diet (no feed additive). According to Lemos et al. (2016), feeding monensin (30 mg/kg of DM) to finishing feedlot cattle resulted in similar growth performance to VM (25 mg/kg DM).

**Table 2.** Effects of feed additives [monensin (**MON**; 30 mg/kg dry matter) or virginiamycin (**VM**; 25 mg/kg dry matter)] and adaptation programs [adding roughage (**ROU**; sugarcane bagasse) or not (**NO-ROU**) during the 20-d adaptation period] on growth performance and carcass characteristics of finishing Nellore bulls fed flint whole shelled corn based diets—Exp. 1

	Feed additives^1^		Adaptation program^2^		SEM^3^	*P*-value		
Item	MON	VM	ROU	NO-ROU		Feed additive	Adaptation program	Feed additive × Adaptation program
Initial body weight, kg	369	369	369	369	6.08	0.98	0.91	0.95
Adaptation period—Days 1 to 20								
Dry matter intake, kg/d	4.42	4.47	4.54	4.36	0.315	0.89	0.57	0.94
Dry matter intake, % of BW	1.18	1.20	1.20	1.18	0.073	0.90	0.72	0.85
Body weight, kg—Day 20	374	379	382	371	6.43	0.53	0.09	0.63
Average daily gain, kg/d	0.268	0.507	0.689	0.098	0.272	0.40	0.05	0.59
Feed efficiency^4^	0.058	0.110	0.152	0.020	0.061	0.35	0.03	0.62
Total feeding period—Days 1 to 105								
Dry matter intake, kg/d	7.22	7.89	7.67	7.44	0.266	0.03	0.41	0.13
Dry matter intake, % of BW	1.63	1.77	1.71	1.69	0.051	0.02	0.72	0.15
Final body weight, kg	518	522	528	512	8.10	0.62	0.08	0.34
Average daily gain, kg/d	1.42	1.47	1.52	1.37	0.075	0.55	0.06	0.33
Feed efficiency	0.198	0.186	0.199	0.184	0.008	0.18	0.09	0.82
Carcass characteristics								
Initial hot carcass weight, kg	197	197	197	197	1.375	0.83	0.96	0.96
Initial dressing percentage	53.5	53.5	53.5	53.5	0.060	0.97	0.88	0.99
Final hot carcass weight, kg	290	292	295	287	5.445	0.73	0.16	0.30
Final dressing percentage	56.0	56.0	56.0	55.9	0.475	0.94	0.80	0.81
Carcass daily gain, kg/d	0.886	0.910	0.937	0.860	0.048	0.63	0.14	0.25

^1^Monensin = Rumensin-200, Elanco Saúde Animal, São Paulo, São Paulo, Brazil; Virginiamycin = Phigrow, Phibro Animal Health Corporation, Guarulhos, São Paulo, Brazil.

^2^During the first 20-d adaption period, bulls fed ROU were progressively stepped up to NO-ROU by decreasing the dietary concentration of sugarcane bagasse every 5 days from 40% to 30% to 20% to 10% (DM basis). At the end of the 20-d adaptation period, bulls fed roughage were then switched to the NO-ROU.

^3^SEM = standard error of mean.

^4^Average daily gain to dry matter intake ratio (G:F).

No differences between MON and VM were observed on carcass characteristics (*P* ≥ 0.18; Table 2). In previous studies (Goodrich et al., 1984; Salinas-Chavira et al., 2009), effects of MON or VM on carcass characteristics were not apparent. Based on the current results, similar growth performance and carcass characteristics should be expected when supplementing MON or VM for finishing cattle fed WC-based diet, and the economic benefit should consider primarily the costs associated with each feed additive.

Adding sugarcane bagasse to WC-based diets during the 20-d adaptation period (ROU) increased the ADG (*P* = 0.05) and the G:F (*P* = 0.03), and tended to increase the BW (*P* = 0.09) compared to NO-ROU (Table 2). During the total feeding period (105 d), bulls fed ROU tended to have greater ADG (*P* = 0.06), final BW (*P* = 0.08), and feed efficiency (*P* = 0.09) compared to NO-ROU (Table 2). It seems that the positive effects of adding roughage during the adaptation period is the main factor affecting growth performance during the total feeding period. observed that According to Marques et al. (2016) adding 3% sugarcane bagasse to a flint WC grain diet increased DMI and ADG of Nellore bulls. According to these authors, the addition of low levels of roughage to the WC-based diets could stimulate mastication (Shain et al., 1999) and increase saliva flow and ruminal motility (Nagaraja and Lechtenberg, 2007), which, in turn, would increase the extent of ruminal fermentation of DM (NASEM, 2016), resulting in greater performance. The digestibility of WC can be influenced by several factors such as cattle age, forage source and level, rumen pH, diet protein source, and content (Gorocica-Buenfil and Loerch, 2005). According to these authors, cattle age and roughage level are the most important factors that can affect WC digestibility. Younger cattle have much greater chewing capacity than older cattle to crack dry corn and increase its nutrient digestibility, and increasing roughage level could increase passage rate and decrease ruminal starch digestibility (Gorocica-Buenfil and Loerch, 2005). According to Turgen et al. (2010), feeding diets containing WC with no added roughage tends to decrease DMI and ADG in finishing steers, but improves feed efficiency and performance calculated dietary net energy compared to steers fed finishing diets containing roughage (4.3–10% DM basis).

No differences on carcass characteristics were observed between ROU and NO-ROU (*P* ≥ 0.14; Table 2), in agreement with previous studies (Utley and McCormick, 1980; Traxler et al., 1995) that also failed to demonstrate an improvement in carcass traits when roughage was added in WC-based diets. A tendency of linear increase in HWC was observed by Marques et al. (2016) when SCB (3% or 6%; DM basis) was added to WC-based diets. In the current trial, although the numerical increase (8 kg) no statistical differences were observed in HCW between ROU and NO-ROU (*P* = 0.16).

No differences in the intake of nutrients (*P* ≥ 0.74; Table 3), total tract apparent digestibility of nutrients and fecal starch (*P* ≥ 0.32; Table 3), and ruminal fermentation characteristics (*P* ≥ 0.15; Table 4) were observed between MON and VM, except for ruminal concentration of isovalerate that tended to be greater for MON than VM (*P* = 0.09; Table 4).

**Table 3.** Effects of feed additives [monensin (**MON**; 30 mg/kg dry matter) or virginiamycin (**VM**; 25 mg/kg dry matter)] on intake and total apparent digestibility of nutrients of finishing steers fed flint whole shelled corn-based diets—Exp. 2

Item	Feed additives		SEM^1^	*P*-value
	MON	VM		
Intake, kg∙animal^−1^∙d^−1^				
Dry matter	5.39	4.98	0.89	0.74
Organic matter	0.72	0.74	0.04	0.76
Crude protein	0.37	0.35	0.06	0.76
Neutral detergent fiber	0.77	0.71	0.13	0.74
Starch	2.10	1.96	0.37	0.79
Total tract apparent digestibility, %				
Dry matter	78.2	75.6	2.96	0.56
Organic matter	81.6	79.4	2.46	0.55
Crude protein	89.6	87.4	1.46	0.32
Neutral detergent fiber	88.6	87.9	2.44	0.85
Starch	59.4	53.3	6.87	0.54
Fecal starch, %	32.2	28.3	3.53	0.44

^1^SEM = standard error of mean.

**Table 4.** Effects of feed additives [monensin (**MON**; 30 mg/kg dry matter) or virginiamycin (**VM**; 25 mg/kg dry matter)] on ruminal fermentation characteristics of finishing steers fed flint whole shelled corn-based diets—Exp. 2

Item	Feed additives		SEM^1^	*P*-value
	MON	VM		
Ruminal pH	5.78	5.83	0.08	0.68
Ruminal ammonia nitrogen, mg/dL	12.4	10.6	1.20	0.32
Total volatile fatty acids, mM	135	142	2.91	0.15
Acetate, mM	69.4	70.9	2.67	0.70
Propionate, mM	40.3	42.3	2.63	0.60
Isobutyrate, mM	1.45	1.71	0.15	0.24
Butyrate, mM	17.3	20.7	2.32	0.33
Valerate, mM	2.86	3.53	0.43	0.31
Isovalerate, mM	4.99	4.21	0.29	0.09
Acetate:propionate ratio	1.88	1.78	0.14	0.65

^1^SEM = standard error of mean.

Monensin is a widely used ionophore that function by selecting against Gram-positive bacteria and protozoa in the rumen (Goodrich et al., 1984). Ionophores can disrupt the ion concentration gradient across microorganism’s membranes causing them to enter a futile ion cycle (Russell and Houlihan, 2003). The most common effect of MON supplementation is the shift in ruminal bacteria. Yang and Russell (1993) reported that when MON is fed, acetate typically decreases or does not change, but propionate concentration increases, lowering the acetate:propionate ratio. Virginiamycin, on the other hand, is a non-ionophore antibiotic produced as a fermentation product of *Streptomyces virginiae* that is also primarily effective against Gram-positive bacteria, particularly lactic acid-producing bacteria (Nagaraja and Taylor, 1987). Virginiamycin acts by blocking protein synthesis through the inhibition of peptide bond formation (Cocito, 1979). The effects of VM on ruminal fermentation and VFA molar proportions has been insignificant (Candanosa et al., 2008; Salinas-Chavira et al., 2009). Therefore, we would expect to detect differences in ruminal fermentation characteristics between MON and VM in the present study due to the different mode of action of each feed additive evaluated herein. The lack of effect is unclear, but is in agreement with the lack of difference in nutrient digestibility coefficients observed in the current study. Zinn et al (1994) and Morris et al. (1990) also did not observe differences in ruminal and total tract digestibility of DM, ADF, and starch of steers supplemented with 28 mg/kg DM of monensin. Montano et al. (2015) and Lemos et al. (2016) also did not observe differences in ruminal fermentation characteristics between steers fed MON and VM. The lack of difference in VFA profile could support the similar growth performance reported in Exp. 1. Similar concentrations of rumen ammonia nitrogen suggest similar efficiency of MON and VM to affect deamination in the rumen.

**Figure 1.**
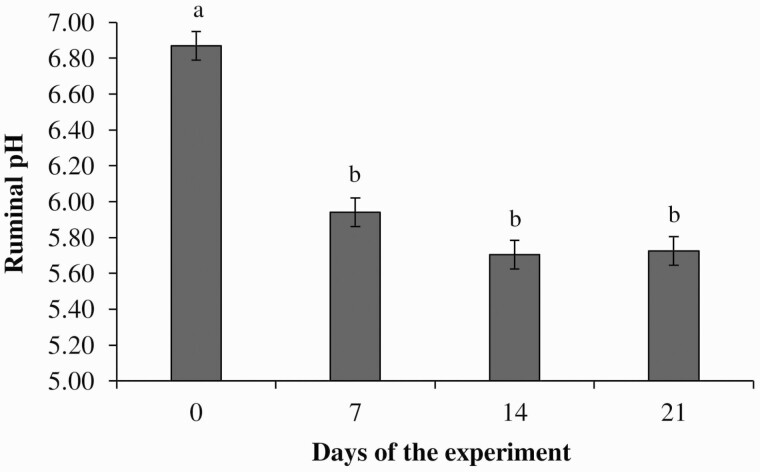
Effects of feed additives [monensin (30 mg/kg of dry matter) or virginiamycin (25 mg/kg of dry matter)] on ruminal pH of Nellore steers fed flint whole shelled corn-based diets. Exp. 2. *P*-values: feed additives, *P* = 0.835; sampling day, *P* ≤ 0.001; interaction between feed additives × sampling day, *P* = 0.336. SEM = 0.075. ^ab^Sampling days with different superscript letters differ (*P* ≤ 0.05; Tukey test).

Ruminal pH between 5.2 and 5.5 (Nagaraja and Titgemeyer, 2007), or below 5.6 for more than 3 h/24 h (Plaizier et al., 2008) can be an indicator of subacute ruminal acidosis. In the current study, the pH was maintained above these values (Table 4 and Figure 1), indicating no metabolic disorders due to feeding no-roughage diets containing flint WC to finishing feedlot cattle.

An effect of sampling day was observed for ruminal pH (*P* < 0.001; Figure 1), which was greater on day 0 compared to day 7, 14, and 21 (*P ≤* 0.05), with no differences between days 7, 14, and 21 (*P* ≥ 0.05; Figure 1). This can be attributed to the diet change at the beginning of the experiment. Steers were moved from the pasture to the feedlot and fed with WC-based diets. The ruminal pH change observed between days 0 and 7 is probably due to the change from grass to the high-concentrate diet. According to Mackie et al. (1978) the proportions of amylolytic and lactate-utilizing bacteria might be expected to increase, and there was likely a shift from acid-sensitive to more acid-tolerant species during the adaptation to high concentrate diets.

## CONCLUSION

Supplementing monensin and virginiamycin for finishing cattle fed whole shelled corn diets resulted in similar growth performance and carcass characteristics, despite a reduction in the dry matter intake for bulls fed monensin. Adding sugarcane bagasse to adapt finishing bulls to no-roughage diets containing whole shelled corn based diets is an alternative to increase growth performance.
